# The Role of Eugenol in the Prevention of Acute Pancreatitis-Induced Acute Kidney Injury: Experimental Study

**DOI:** 10.1155/2016/3203147

**Published:** 2016-01-17

**Authors:** Charalampos Markakis, Alexandra Tsaroucha, Apostolos E. Papalois, Maria Lambropoulou, Eleftherios Spartalis, Christina Tsigalou, Konstantinos Romanidis, Constantinos Simopoulos

**Affiliations:** ^1^Department of Surgery, St. Georges' Hospital, Tooting SW17 0QT, UK; ^2^Department of Surgery and Laboratory of Experimental Surgery, Faculty of Medicine, Democritus University of Thrace, 68100 Alexandroupolis, Greece; ^3^Experimental Research Center, ELPEN Pharmaceuticals, Pikermi, 19009 Attica, Greece; ^4^Laboratory of Histology, Faculty of Medicine, Democritus University of Thrace, 68100 Alexandroupolis, Greece; ^5^Second Propaedeutic Department of Surgery, Thoracic Surgery Department, “Laiko” General Hospital, 11527 Athens, Greece

## Abstract

*Aim*. Acute pancreatitis is an inflammatory intra-abdominal disease, which takes a severe form in 15–20% of patients and can result in high mortality especially when complicated by acute renal failure. The aim of this study is to assess the possible reduction in the extent of acute kidney injury after administration of eugenol in an experimental model of acute pancreatitis.* Materials and Methods*. 106 male Wistar rats weighing 220–350 g were divided into 3 groups: (1) Sham, with sham surgery; (2) Control, with induction of acute pancreatitis, through ligation of the biliopancreatic duct; and (3) Eugenol, with induction of acute pancreatitis and eugenol administration at a dose of 15 mg/kg. Serum urea and creatinine, histopathological changes, TNF-*α*, IL-6, and MPO activity in the kidneys were evaluated at predetermined time intervals.* Results*. The group that was administered eugenol showed milder histopathological changes than the Control group, TNF-*α* activity was milder in the Eugenol group, and there was no difference in activity for MPO and IL-6. Serum urea and creatinine levels were lower in the Eugenol group than in the Control group.* Conclusions*. Eugenol administration was protective for the kidneys in an experimental model of acute pancreatitis in rats.

## 1. Introduction

Acute pancreatitis is an inflammatory intra-abdominal process, which in approximately 15–20% of patients presents in a severe form, with a gradual establishment of multiple organ dysfunction or local complications, including necrosis, pseudocyst, and abscess [1]. Severe acute pancreatitis is a condition associated with high mortality, which is characterized by a complex and incompletely understood pathophysiological mechanism [2, 3].

The deficit in our understanding of the mechanism driving the inflammatory process in acute pancreatitis is a reason why our therapeutic strategy has failed to reduce mortality, despite ongoing research. The aetiology of early mortality after acute pancreatitis is multiple organ failure. When acute pancreatitis leads to the establishment of acute kidney injury, there is a 5- to 10-fold rise in mortality, which can reach 70% [4–6]. The prevention of acute kidney injury can be a useful strategy in the prevention of the morbidity and mortality associated with acute pancreatitis.

Eugenol (1-allyl-4-hydroxy-3-methoxybenzene) is a naturally occurring substance, found in the essential oil of commonly consumed spices such as clove oil as well as cinnamon, basil, and nutmeg oils [7]. It has many pharmacological properties which are mainly analgesic, anti-inflammatory, antioxidant, and vasodilatory action [7], while it has been shown to ameliorate kidney injury in a model of gentamycin-induced nephrotoxicity [8]. The aim of this study is to assess the possible reduction in the extent of acute kidney injury after administration of eugenol in an experimental model of acute pancreatitis.

## 2. Materials and Methods

### 2.1. Experimental Animals

106 male Wistar rats, aged 3-4 months and weighing 220–350 gr, were used in this study. They were housed in cages under standard laboratory conditions (12 hr light-dark cycles, 22–25°C room temperature, and 55–58% humidity), with free access to food and water. The animals were procured from the Hellenic Pasteur Institute (Athens, Greece). The experiment took place at the ELPEN Experimental Research Center (Pikermi, Greece), while the histological analysis was carried out at the Lab of Histology, Embryology, Medical School, Democritus University of Thrace. The experimental surgical procedures and the general handling of the animals conformed to the international guidelines of Directive 86/609/EEC on the protection of animals used for experimental and other scientific purposes. The animals were randomly assigned in 3 groups: Sham (*N* = 20), Control (*N* = 46), and Eugenol (*N* = 40).

### 2.2. Acute Pancreatitis Experimental Model

The animals were anaesthetized initially by being placed in a glass box containing isoflurane and then through administration of 0.25 mL of butorphanol (Dolorex; Intervet/Schering/Plough Animal Health, Boxmeer, Holland) by subcutaneous injection. The animals were intubated with a 16 G venous catheter, which was then connected to a ventilator set at 70 breaths/min and a tidal volume of 3 mL. After confirmation of the success of intubation, anaesthesia was maintained by a mixture of 93% O_2_, 5% CO_2_, and 2% isoflurane. Acute pancreatitis was induced according to a previously described model [9]. Briefly, after induction of anaesthesia and preparation of the surgical site, the abdomen was entered via a 3 cm midline incision under sterile conditions. The pancreas was identified and mobilized in all animals. The biliopancreatic duct was identified and ligated near the duodenal wall with a 4-0 silk sutures (in the Control and Eugenol groups, but not in the Sham group). 1 mL of normal saline and 1 mL of 5% D_5_W were instilled in the abdominal cavity. The abdomen was closed with vicryl 2-0 sutures. In the Eugenol group, eugenol was administered by a nasogastric catheter in a dose of 15 mg/kg, while the Sham and Control groups received corn oil solution without eugenol.

Postoperatively, analgesia was maintained through subcutaneous administration of 2 mL/Kg butorphanol (Dolorex; Intervet/Schering/Plough Animal Health, Boxmeer, Holland). Euthanasia was performed at a predetermined time for each animal with the use of ketamine (Narcetan; Vetoquinol, Buckingham, UK) 0.3–0.6 mL and xylazine (Rompun; Bayer, Uxbridge, UK) 0.1–0.3 mL, followed by a midline laparotomy and exsanguination of the abdominal aorta. Time points for analysis were 6, 12, 24, 48, and 72 hours postoperatively. Serum samples for measurement of urea and creatinine as well as specimens from both kidneys for histopathological examination were acquired.

### 2.3. Preparation of Eugenol

Pure eugenol (eugenol 99%, Aldrich Chemistry, St. Louis, MO, USA) was purchased and prepared in an oily solution in the chemical laboratory of Elpen Pharmaceutical Co. Inc. (ELPEN Pharmaceutical Co. Inc., Pikermi Attica, Greece). This was achieved with the admixture of pure eugenol in a corn oil solution in a concentration of 1.5 mg eugenol/mL.

### 2.4. Histopathological and Immunohistochemical Evaluation

Samples were placed in 10% buffered formalin solution, and 4 *μ*m paraffin-embedded sections were stained with hematoxylin/eosin. All specimens were evaluated by a pathologist blinded to the sequence of the biopsy specimens. Slides were evaluated with regard to 5 histopathological parameters and with the use of a semiquantitative scoring system as depicted on Table 1. The scores of each individual parameter for each slide were added and a histopathological score was obtained for each specimen.

Immunohistochemical staining was applied to detect the possible expression of inflammatory cytokines like IL-6, TNF-*α*, and myeloperoxidase. The following antibodies were used: myeloperoxidase (rabbit polyclonal), DAKO (A 0398), diluted 1 : 400 TNF-*α* (rabbit polyclonal), ABNOVA (PAB8016), diluted 1 : 1000, IL-6 (rabbit polyclonal), and Abcam (ab6672), diluted 1 : 500.

The buffers, blocking solutions, secondary antibodies, avidin-biotin complex reagents, and chromogen were supplied in a detection kit (EnVision HRP, Mouse/Rabbit detection system (K 5007), DAKO). To inhibit endogenous peroxidase, the specimens were incubated with 3% H_2_O_2_ (200 mL H_2_O and 6 mL H_2_O_2_) for 15 min in a dark room. Before the primary antibody was applied, the sections were immersed in 10 mM citrate buffer (pH 6.0), rinsed in tris-buffered saline, and subsequently heated in a microwave oven (650–800 W) for three cycles of 5 min. The slides were washed with tris-buffered saline before application of the primary antibody in order to reduce nonspecific binding of antisera. Control slides were used as common negative controls for all antibody staining. Sections were then briefly counterstained with Mayer's hematoxylin, mounted, and examined under a Nikon Eclipse 50i microscope (Nikon Instruments Inc, NY, USA).

Scoring was assigned according to the proportion of cells with cytoplasmic staining. The positivity of the expression was determined by counting the number of stained cells. The average labeling index was assessed according to the proportion of positive cells, after scanning the entire section of the specimen. Sections with greater than 10% stained cells were considered as being positive. The results were graded as negative (0) for <10% of stained cells, low (1) for >10% and <30% of cells stained, moderate (2) for >30% and <70% cells stained, and high expression (3) for >70% cells stained (Table 1).

### 2.5. Statistical Analysis

The statistical analysis of the results was completed with the use of the 20th version of SPSS (Statistical Package for the Social Sciences, SPSS Inc., Chicago, IL, USA). We performed an analysis in which the data were treated as qualitative using Fisher's exact test (this test was preferable to *x* ^2^ because of the small number of animals in each subcategory/time point). Use of the semiquantitative scoring allowed us to also treat the data as ordinal. Evaluation of the different variables was performed to determine whether they were normally distributed (Kolmogorov-Smirnov are Shapiro-Wilk). The three different groups were then analyzed using the Kruskal-Wallis one-way analysis of variance test. Finally, the Mann-Whitney *U* test was further used to compare the groups in pairs. These tests were applied to the overall sample and for each individual subgroup corresponding to individual time points (6, 12, 24, 48, and 72 hours postoperatively).

## 3. Results

### 3.1. Surgical Outcomes

The operation was concluded successfully on all animals and all animals survived the operation. The animals resumed normal diet and activity with normal bowel function. Six animals died before the predetermined time point for their euthanasia. These animals were all part of the Control group. They were substituted and were not included in the statistical analysis.

### 3.2. Statistical Analysis

The statistical analysis showed that none of the variables followed a normal distribution. Thus, nonparametric tests were used to analyze the results.

### 3.3. Histological Evaluation

**Figure 1:**
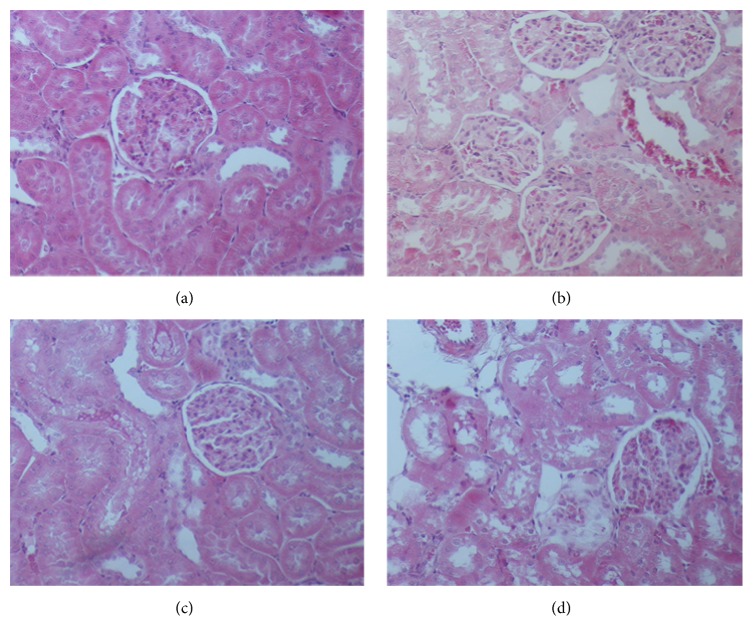
Different levels of inflammation and the corresponding histopathological score (H&E, ×200). (a) Sham group: score 0. (b) Control group: score 3. (c) Eugenol group: score 5.5. (d) Control group: score 9.

**Figure 2:**
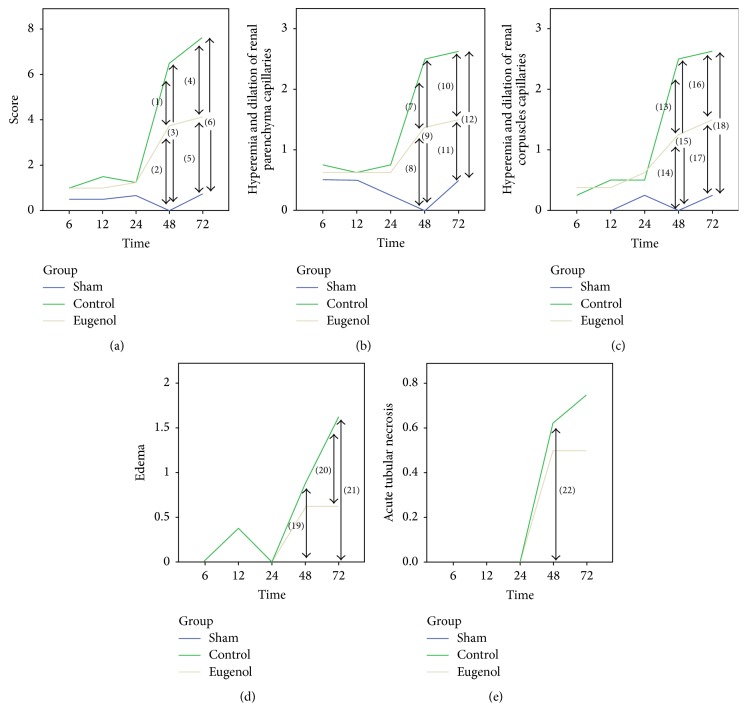
Comparative results of histopathological analysis (statistically significant differences are marked by arrows). (a) Histological score. (b) Hyperemia and dilatation of renal parenchymal capillaries. (c) Hyperemia and dilatation of renal corpuscles capillaries. (d) Edema. (e) Acute tubular necrosis. *p* values (Mann-Whitney test) (1): 0.002, (2): 0.004, (3): 0.004, (4): <0.001, (5): 0.004, (6): 0.004, (7): 0.005, (8): 0.004, (9): 0.004, (10): 0.005, (11): 0.048, (12): 0.004, (13): 0.002, (14): 0.004, (15): 0.004, (16): 0.005, (17): 0.016, (18): 0.004, (19): 0.048, (20): 0.007, (21): 0.004, and (22): 0.048.

Eugenol administration resulted in a lower histological score in rats with acute pancreatitis. The difference between the Eugenol and Control groups is apparent at 48 and 72 hours after induction of pancreatitis (Figures 1 and 2). The histological score for these two groups is higher compared to the Sham group at 48 and 72 hours and for the whole sample.

Eugenol administration lowers hyperemia and dilation of renal parenchyma capillaries and the difference was statistically significant for the 48 and 72 hour time points and for the whole sample. The Eugenol group exhibited lower values than the Control group and both exhibited higher values than the Sham group. The same was true for hyperemia and dilation of renal corpuscles capillaries for the 48- and 72-hour time points, but not for the whole sample.

There were no inflammatory infiltrations in any of the animals in our experimental model and measurement of this factor did not produce any results.

Edema was reduced through the administration of eugenol and, again, the difference to the Control group was significant for the 48- and 72-hour time points and the whole sample. The Control group had higher values than the Sham group at 48 hours and also higher values than both the Sham and Eugenol groups at 72 hours. When values of the whole sample were considered, the Control group had higher values than the Eugenol group, which in turn had higher values than the Sham group.

Finally, eugenol did not reduce acute tubular necrosis in our experimental model. There was no statistically significant difference at any of the time points studied. Analysis of the whole sample showed only higher values for the Eugenol and Control groups when compared to the Sham group.

### 3.4. Immunohistochemical Evaluation

**Figure 3:**
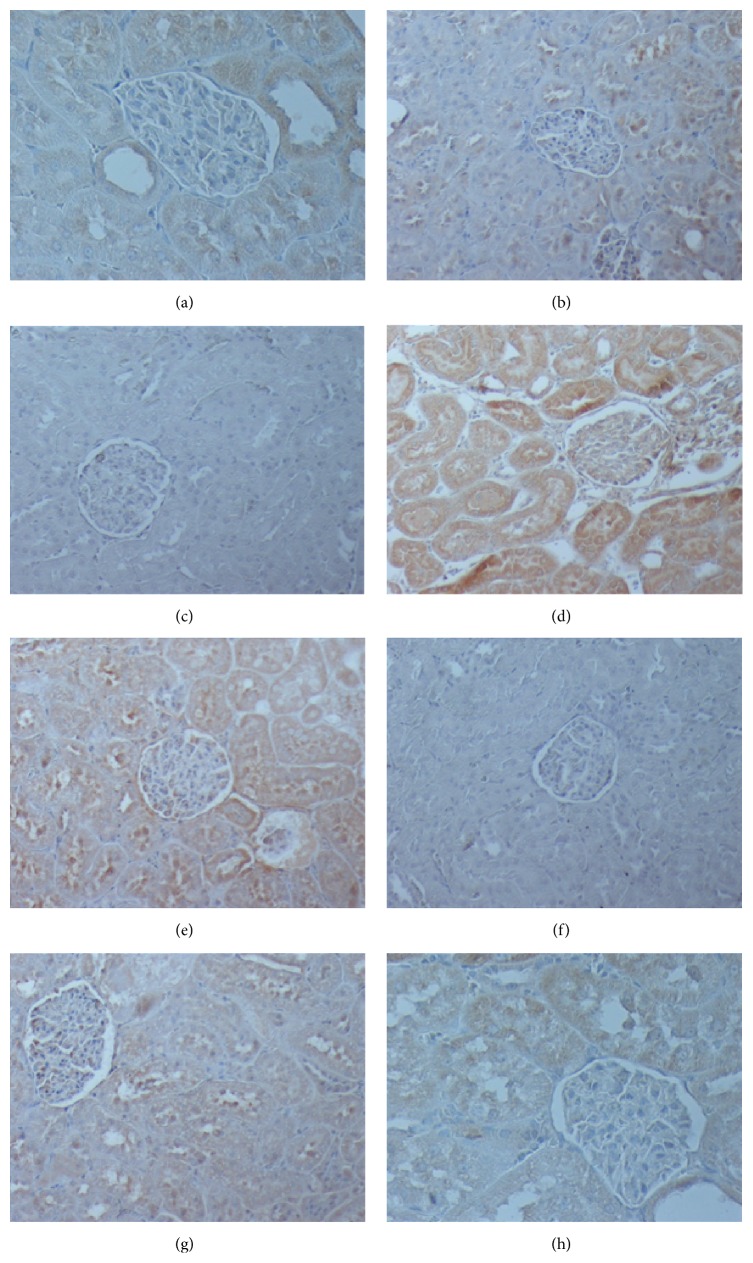
Different levels of immunohistochemical staining expression (×200). (a) Control group: mild expression of IL-6 after 72 hours. (b) Eugenol group: moderate expression of IL-6 after 72 hours. (c) Sham group: no expression of TNF-*α* at 72 hours. (d) Control group: moderate expression of TNF-*α* at 72 hours. (e) Eugenol group: mild expression of TNF-*α* at 72 hours. (f) Sham group: no expression of MPO at 72 hours. (g) Control group: severe expression of MPO at 72 hours. (h) Eugenol group: moderate expression of MPO at 72 hours.

**Figure 4:**
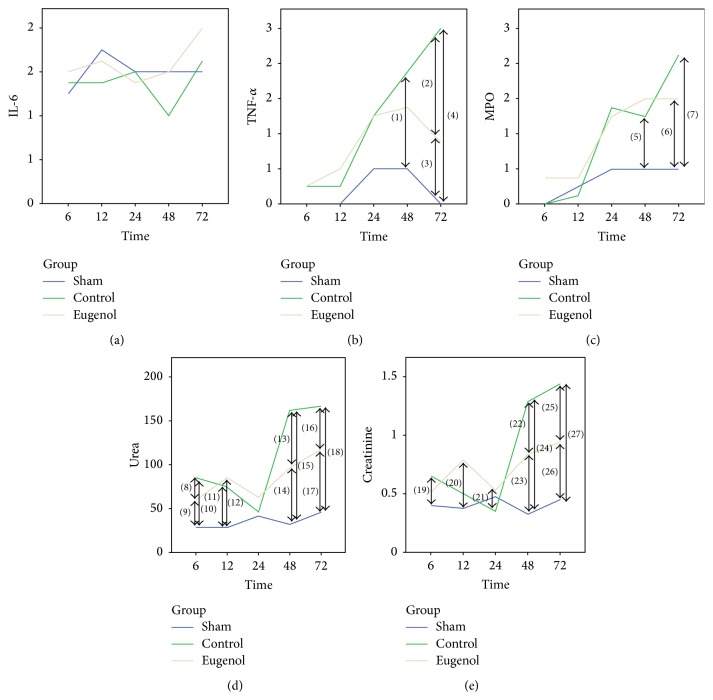
Serum urea and creatinine and immunohistochemical staining results (statistically significant differences are marked by arrows). (a) IL-6. (b) TNF-*α*. (c) MPO. (d) Urea. (e) Creatinine. *p* valves (Mann-Whitney test) (1): 0.008, (2): <0.001, (3): 0.016, (4): 0.004, (5): 0.048, (6): 0.008, (7): 0.048, (8): 0.050, (9): 0.004, (10): 0.004, (11): 0.004, (12): 0.004, (13): <0.001, (14): 0.004, (15): 0.004, (16): 0.001, (17): 0.004, (18): 0.004, (19): 0.008, (20): 0.028, (21): 0.001, (22): 0.040, (23): 0.004, (24): 0.004, (25): 0.002, and (26): 0.004.

There was no clear difference regarding IL-6 expression between the different groups (Figures 3 and 4). On the contrary, TNF-*α* expression was attenuated through eugenol administration. There was a statistically significant difference between the Eugenol and Control groups 72 hours after induction of pancreatitis, while both groups exhibit higher TNF-*α* expression than the Sham group. There was no statistically significant difference between the Eugenol and Control groups for MPO expression, although there was a trend toward higher expression for the Control group after 72 hours.

### 3.5. Renal Function

Eugenol administration resulted in lower serum levels of urea and creatinine especially at the 48- and 72-hour time points, compared to the Control group. Urea and creatinine levels were higher for both the Eugenol and Control groups, when they were compared to the Sham group (Figure 4).

## 4. Discussion

The results of this study suggest that eugenol attenuates the intensity of the histopathological changes and the expression of TNF-*α* and MPO in the renal parenchyma, while lowering the values of serum urea and creatinine when administered in a rat acute pancreatitis experimental model.

To evaluate the extent of kidney injury, we decided to evaluate serum urea and creatinine levels and the histopathological changes in the kidney, as well as the expression of TNF-*α*, IL-6, and MPO in the renal parenchyma. The role of cytokines, such as TNF-*α* and IL-6, in the pathophysiology of acute pancreatitis has been studied extensively and they have been found to contribute to the activation of the systematic inflammatory response process and multiorgan failure, which is a hallmark of severe acute pancreatitis and is, ultimately, correlated with the observed high mortality rates [10, 11]. The role of cytokines in acute kidney injury has been found to be equally important. The cytokine-mediated inflammatory response has a central role in the pathophysiology of acute renal failure irrespective of its cause. MPO has been used as a marker of neutrophil migration in acute pancreatitis studies and has been correlated to the severity of kidney injury [12–14].

The histopathological evaluation showed that the histologic score was lower for the Eugenol group in comparison to the Control group at 48 and 72 hours from the initiation of the inflammatory process (means: 3.75/6.5 and 4.12/7.62, resp.) and this difference was statistically significant. This difference between the two groups was also present for individual histological changes such as hyperemia and dilation of renal parenchyma and renal corpuscles capillaries and edema. The difference observed in the degree of acute tubular necrosis and inflammatory infiltration was not statistically significant.

Regarding the expression of inflammatory mediators, TNF-*α* levels were higher for the Control group in comparison to the Eugenol group with the difference reaching statistical significance at the 72-hour time point, while there was a trend for higher MPO expression in the Control group at 72 hours, which was, however, not statistically significant. In contrast, IL-6 levels did not show the same correlation and there were no statistically significant differences between the Eugenol and Control groups.

We chose the bile-pancreatic duct ligation model as it is a well-characterized model of acute pancreatitis, which mimics acute pancreatitis caused by biliary obstruction, which is a frequent clinical scenario and results in multiorgan failure similar to that observed in humans [15, 16]. We have previously used this experimental model and we were able to show that it generates acute pancreatitis with histopathological changes in the pancreatic tissue including hemorrhage and necrosis [17]. Out of a total of 106 animals, 6 died and the fact that they were all in the Control group could be seen as further evidence supporting the protective role of eugenol. It is possible that these animals would have exhibited signs of severe kidney injury, if they had survived until the predetermined time of euthanasia. However, since the distal bile-pancreatic duct ligation model is not usually fatal, we cannot directly attribute the death of these animals to the severity of acute pancreatitis. These animals were, therefore, excluded from the statistical analysis and were replaced.

Eugenol has been shown to possess a multitude of pharmacological effects [7], some of which make it a likely candidate for use in the setting of acute pancreatitis and can explain the results observed in our study. The analgesic action of eugenol has been well documented and doses in the range of 40–100 mg/kg have been shown to be effective in rat experimental models [18–20]. In addition, eugenol acts as an anti-inflammatory substance inhibiting cyclooxygenase [21] and reducing the release of proinflammatory mediators such as IL-1*β*, TNF-*α*, and PGE2 [22–24]. The antioxidative potential of eugenol has been studied in a number of, mainly in vitro, studies where it has been shown to bind to free oxygen radicals and attenuate the action of oxidative substances [25–28], while a recent study of gentamycin-induced nephrotoxicity offers insight into how eugenol can prevent kidney injury by reducing oxidative damage [8]. These combined properties of eugenol can be used to explain the observed reduction in TNF-*α* expression, as well as the reduction of kidney inflammation. Eugenol administration causes a dose-dependent, reversible vasodilation through its effect on the endothelial cells [29, 30], which is comparable to nifedipine [31]. The potential of eugenol to inhibit the vasoconstriction that is associated with kidney injury points to another potential mechanism for its effect in the model of acute pancreatitis.

A number of authors have proposed strategies to reduce kidney injury caused by acute pancreatitis. Zhang et al. have tried dexamethasone administration in an experimental model of retrograde injection of sodium taurocholate in the pancreatic duct [32]. The dexamethasone group exhibited milder congestion of the glomerular capillary, swelling of the renal tubular epithelial cells, and less inflammatory cell infiltration than that of the Control group, which was shown by the lower histological score at the 6- and 12-hour time points. The same authors found a significant difference in the serum levels of TNF-*α* in favor of the dexamethasone group, while expression of NF-*κ*B in the renal tissue was more pronounced in the dexamethasone group [33]. The same model has been used to study octreotide and baicalin (5,6,7-trihydroxyflavone-7-O-D-glucuronic acid) [34]. The administration of these substances had a protective effect on the kidney and both the histological score and renal parenchyma NF-*κ*b expression were lower in comparison to the Control group. Serum levels of urea, creatinine, TNF-*α*, and IL-6 were reduced compared to the Control group in another study with the same experimental protocol [35]. There have been a number of studies of plant derived substances, used in traditional Chinese medicine. Ligustrazine proved to be protective for the kidney as was demonstrated by the lower creatinine levels and the milder histopathological changes in comparison to the Control group [36]. In another study, the administration of 3 traditional Chinese medicine substances (ligustrazine, kakonein, and* Panax* notoginsenosides) resulted in reduced mortality and milder histopathological changes in the rat kidney [37]. Finally, the model of induction of acute pancreatitis through sodium taurocholate administration was used for the study of poly(ADP-ribose) polymerase inhibition, through 3-aminobenzamide (3-AB) administration. The administration of 3-AB resulted in reduced mortality and a reduction in the increase of creatinine, TNF-*α*, IL-1b, and IL-6, milder histopathological changes, and reduced MPO expression in the kidney [18].

There are some limitations to our experimental protocol. The half life of eugenol in the rat has been determined to be 18,3 hours [18]; therefore, at 72 hours, most of the initial dose would have been cleared from the circulation. It is possible that a repeat administration of eugenol could further increase the therapeutic result. Moreover, the time frame of our protocol reached 72 hours, which was not adequate for the complete evaluation of the effect of eugenol. Indeed, a difference in the extent of kidney injury between the Eugenol and Control groups is first observed 48 hours after the onset of acute pancreatitis and it is greater at 72 hours. Further observations at additional time points could yield even larger differences in results.

In conclusion, the administration of eugenol in a rat model of acute pancreatitis was protective for the kidneys in our experimental model. Further research is necessary to determine the possible role of eugenol in the management of acute pancreatitis.

## Figures and Tables

**Table 1:** Semiquantitative scoring system.

Variable	Result	Score
Histopathological variables		
Hyperemia and dilation of renal parenchyma capillaries	None	0 = −
Hyperemia and dilation of renal corpuscles capillaries	Mild	1 = +
Inflammatory infiltration of renal parenchyma		
Edema	Moderate	2 = + +
Acute tubular necrosis	Severe	3 = + ++

Immunohistochemical variables		
IL-6	None	0 = −
TNF-*α*	Mild	1 = +
	Moderate	2 = + +
MPO	Severe	3 = +++
